# Critical thinking skills of nursing students: Observations of classroom instructional activities

**DOI:** 10.1002/nop2.426

**Published:** 2019-11-22

**Authors:** Christian Makafui Boso, Anita S. van der Merwe, Janet Gross

**Affiliations:** ^1^ Department of Nursing and Midwifery Faculty of Medicine & Health Sciences Stellenbosch University Cape Town South Africa; ^2^ School of Nursing and Midwifery College of Health and Allied Sciences University of Cape Coast Cape Coast Ghana; ^3^ Peace Corps Liberia Mother Patern College of Health Sciences Stella Maris Polytechnic Monrovia Liberia

**Keywords:** classroom, critical thinking, learning environment, nurse educator, nursing student

## Abstract

**Aim:**

Critical thinking (CT) is vital for nursing practice. Nursing schools should provide learning experiences that enable nursing students to acquire CT skills. Yet, these authors are not aware of any study that has directly observed instructional activities related to CT skills acquisition in the classroom environment. The aim of this study was to explore instructional activities in the classroom environment in relation to acquisition of CT skills of students.

**Design:**

Qualitative non‐participant observation.

**Methods:**

Using a purposive sampling, 10 classroom teaching sessions were observed and mediating factors of CT skills acquisition of students noted. Data were analysed thematically. Data were collected from October–December 2017.

**Results:**

Three key themes of instructional activities relating to acquisition of CT skills of students emerged, namely educators’ behaviour, students’ characteristics and university‐wide factors/administrative support. Class sizes ranged from 34–162 students with an average of 95.

## 1 INTRODUCTION

The ever‐changing and complex healthcare environment requires that nurses acquire critical thinking (CT) skills to meet the complex challenges of the environment (Von Colln‐Appling & Giuliano, 2017). Nurses should be able to select and use data for effective clinical judgements to promote good health outcomes (Nelson, 2017; Von Colln‐Appling & Giuliano, 2017). Consequently, nursing schools must offer learning experiences that assist students to think critically about complex issues instead of just merely becoming receptacles for information (Toofany, 2008; Von Colln‐Appling & Giuliano, 2017). It is the duty of nurse educators to help students to acquire CT skills (Nelson, 2017; Von Colln‐Appling & Giuliano, 2017).

Attempts have been made to conceptualize CT to guide the facilitation of CT skills of students. Worth noting are Dwyer, Hogan, and Stewart (2014) and Duron, Limbach, and Waugh’s (2006) frameworks, which could be relevant in the classroom setting. Focusing on learning outcomes, Dwyer et al. (2014) posited that long‐term memory and comprehension are foundational processes for CT application. The framework incorporates both reflective judgement and self‐regulatory functions of metacognition as a requirement for CT. Self‐regulation refers to an individual's ability, willingness and the perceived need to think critically when solving specific problems. Therefore, factors that influence the interrelationship between short‐term and long‐term memory (the bedrock of CT), comprehension, reflective judgement and self‐regulation functions of metacognition will influence CT skills of the students. On the other, Duron et al.’s model focused on practical instructional activities needed to guide students in acquiring CT skills. The five‐step framework requires that educators: (a) determine learning objectives; (b) teach through questioning; (c) practice before assessing; (d) review, refine and improve; and (e) provide feedback and assessment of learning.

Nursing literature is replete with studies demonstrating that adopting appropriate teaching methods/strategies, such as active learning, improves the CT scores of students. Examples of such approaches include problem‐based learning (Jones, 2008; Jun, Lee, Park, Chang, & Kim, 2013), concept mapping (Wheeler & Collins, 2003) and simulation (Sullivan‐Mann, Perron, & Fellner, 2009). Furthermore, based on a systematic review, Chan (2013) suggested three strategies to facilitate CT skills of nursing students, which include appropriate questioning strategy, reflective writing on learning experiences and discussion of case study.

The classroom environment provides a vital opportunity for educators to create the necessary milieu to encourage students to develop their CT skills. It is therefore required that negative factors to the development of CT are minimized or removed and those factors that enhance the development of CT skills are accentuated. However, these factors that influence CT have received less attention in nursing education (Raymond, Profetto‐McGrath, Myrick, & Strean, 2018). Furthermore, no direct observations have been made to identify specific factors influencing CT in the classroom setting.

Studies such as those of Mangena and Chabeli (2005) and Shell (2001) assessed factors that inhibit CT acquisition of nursing students. Mangena and Chabeli's study focused on educators and students’ perspectives. They found that educators’ lack of knowledge of CT teaching methods and evaluation, negative attitudes of educators, student selection and educational background, socialization, culture and language inhibited the development of CT skills of students. Shell also found negative student factors, demand to cover content and time constraints both on class time and on educators’ development that hindered CT skills development of students.

Raymond and Profetto‐McGrath (2005) also identified internal and external factors of educators that had an impact on their CT. These factors included physical and mental well‐being, the view of leadership on CT and collegial relationships that existed in the educators’ environment. Similarly, Raymond et al. (2018) identified personal (elements/conditions originating from the educator), interpersonal (elements originating from the student–educator relationship) and broader environmental factors (conditions evident in the larger physical setting or political milieu) that influenced educators’ CT and influenced their abilities to role model CT skills.

The above authors focused on factors influencing CT from different perspectives. Shell (2001) and Mangena and Chabeli (2005) focused on barriers to student development of CT. Also, Shell examined educators’ perspectives. Mangena and Chabeli examined both educators’ and students’ views. Raymond and Profetto‐McGrath (2005) and Raymond et al. (2018) specifically focused on nurse educators' CT skills. None of the above studies directly observed classroom teaching though similar factors were identified.

## 2 BACKGROUND

The “greatest healthcare resource is the healthcare personnel, of which nurses are a primary component” (Talley, 2006, p. 50). However, limited resources in nursing schools especially in developing countries where this study was undertaken (Talley, 2006) impede the experiences required for the students to develop CT skills. For example, studies have identified lack of qualified educators (Bell, Rominski, Bam, Donkor, & Lori, 2013; Salifu, Gross, Salifu, & Ninnoni, 2018) as well as infrastructural and logistical constraints (Talley, 2006), large class sizes and absenteeism (Wilmot, Kumfo, Danso‐Mensah, & Antwi‐Danso, 2013) as some of the challenges affecting nursing education. These challenges have led to the dominance of inappropriate teaching approaches (Boso & Gross, 2015; Wilmot et al., 2013).

Similarly, studies regarding CT have reported the negative influence of sociocultural norms such as the seniority tradition (Chan, 2013; Jenkins, 2011; Kawashima, 2003; Mangena & Chabeli, 2005). In such cultures, students are not encouraged to speak out openly (Chan, 2013). For example, an individual is not expected to disagree nor question an authority figure in public. In the context of this study, the seniority tradition could have been manifested in the classroom where the faculty is regarded as an authority whose ideas may be seen as sacrosanct by students. These authors argue that it is necessary to identify the factors through direct observation that might hinder or enhance the facilitation of CT of students in the classroom setting. Notwithstanding, the authors of this paper had not found any publication in the nursing literature where direct observation for CT teaching methods/strategies had been carried out in the classroom setting. Therefore, this study explored factors that might influence students’ ability to memorize and comprehend content towards CT skills acquisition. Also, educators’ instructional activities that either enhanced or inhibited students’ CT facilitation in the classroom context were explored.

## 3 THE AIM OF THE STUDY

The aim of this study was to explore instructional activities towards the development of CT skills of students in a classroom environment. This study was part of a larger research project aimed at developing a CT‐based curriculum framework of students.

## 4 RESEARCH DESIGN

Qualitative non‐participant observation design was used. This design was to allow for the observation of first‐hand (Patton, 2015) and unusual aspects (Creswell & Creswell, 2018) real‐time classroom practices whilst being present. Also, qualitative observation has been noted as a primary means of understanding the experiences of users (Reddacliff, 2017).

## 5 METHOD

### 5.1 Setting

The study was conducted in classroom settings of an undergraduate nursing programme in a public university in Ghana. As a school in a developing country, there are constraints such as logistical inadequacies and lack of adequate qualified faculty, which could inhibit meaningful learning experience towards CT skills development of students existed. The classes are scheduled based on the demands of the various departments of the university. The university runs several programmes, and each programme is allocated with venues as demanded.

### 5.2 Sampling

Through a purposive method ten (10) teaching sessions from class levels 200 to 400 were observed from October to December 2017. Educators who had lectures within the period were approached face‐to‐face. Ten out of 16 educators agreed to participate. They provided informed consent. The 10 sessions provided rich data to be able to deduce current practices of instruction as occurring in the classroom environment. The main selection criterion was a full teaching session (1–3 hr) of B.Sc. nursing undergraduate programme taught by an educator in the selected nursing school.

### 5.3 Data collection and instrumentation

Data were collected between October and December 2017. The observations were from five level 200, three level 300 and two level 400 classes; six medical–surgical, one maternal health, one biomedical and two nursing fundamental/theoretical courses were taught. Two individuals—first author and an assistant, consistent with Winter and Munn‐Giddings’ (2001) recommendation for observation, observed the teaching sessions. A six‐item semi‐structured observation guide/protocol using Billing and Halstead's (2005) six steps of designing learning experiences for developing CT skills was employed for data collection. Billing and Halstead's six steps of designing experiences for developing CT skills were consistent with identifying factors that enhance or inhibit memory, comprehension, reflective judgement and instruction identical to Dwyer et al. (2014) and Duron et al.’s (2006) frameworks. The protocol was pre‐tested in a classroom at an analogous nursing school. Though the sixth step of Billing and Halstead's (2005) six steps of designing learning experiences for developing CT skills proposes both summative and formative assessments, in the context of this observation, only formative assessment methods used by the educators could be observed.

Billing and Halstead's six steps of designing experiences for developing CT skills are as follows. Step 1 involved determining the learning outcome for the specific class. These learning objectives should be explicitly clear to students and fit for purpose. Step 2 involved creating an anticipatory set. The educator's strategies that generate students’ interest in content, encourage their participation and create collegial environment for students were observed. Step 3 consisted of selecting teaching and learning strategies. Observation focused on identifying active learning methods of teaching against passive teaching methods. Also, whether the educator or students dominated the class was explored. Whether the nurse educator combined different teaching methods/strategies were explored. Step 4 considered implementation issues. Class size, involvement of students, classroom arrangement, use of teaching aids and materials and instructional media were observed. Step 5 involved the observation of how the learning experience was closed. This included how the educator summarized the lesson and related lessons to next class period. Step 6 involved how students’ learning experiences were evaluated. The educator's strategies for the assessment and evaluation of student learning experience during class period were observed.

The observers positioned themselves at the back of the classrooms throughout each period of teaching. Participants did not appear distracted or uncomfortable during the periods of observation. Thoughts and feeling of the observers relative to observed situations were captured as field notes. In order not to distract and cause discomfort to participants, the observers took minimal notes and expanded them immediately after the observations. Transcripts from the observations were compared and agreed on by the two observers. Differences were resolved through discussion. Also, the educators whose teaching sessions were observed were asked to provide feedback and revision made based on educators' comment(s). This was to minimize observers’ bias.

### 5.4 Data analysis

Bryman's (2010) four stages of qualitative analysis as described by Gibbs (2010) were used to analyse the data. The first author and an assistant described each observation. Later, the first author read the transcript at least four times to enable a meaningful content analysis. Data were coded, and themes and sub‐themes were derived. Subsequently, the second and third authors who are the supervisors of this research project cross‐checked the themes and sub‐themes with the observational transcripts for validation.

### 5.5 Ethical consideration

This study was approved as one part of a doctoral project by the Health Research Ethics Committee of Stellenbosch University (Ref. no S17/05/106) and the university where the study was done. Permission was also sought from the dean of the selected school. The first author visited the students at their various classrooms to explain the nature and purpose of the study to them. Likewise, the nurse educators were provided with information on the purpose and nature of the study. They were provided individually with informed consent forms for signing before data were collected. They were assured of their rights to opt out at any stage of the study. Confidentiality and anonymity were also assured. Individual participants were not identified with the data (during data collection, analysis and reporting).

## 6 RESULTS

Three overall themes were deduced from the classroom observation data, namely educators’ behaviour, students’ behaviour and university‐wide factors/administrative support. These themes related to the Dwyer et al. (2014) and Duron et al.’s (2006) frameworks of CT development. To reiterate, these factors could either enhance or inhibit memory (foundation of CT development), comprehension, reflective judgement and self‐regulatory functions of metacognition as a requirement for CT.

### 6.1 Theme I: Educators’ classroom behaviour

Educators’ behaviour includes actions and inactions of the educators that might either enhance or inhibit students’ positive learning experiences towards the acquisition of CT skills. Four sub‐themes under this theme were identified namely beginning and ending on time; creating a conducive and participatory environment; and teaching methods and styles and managing the class.

#### 6.1.1 Subtheme A: Beginning and ending on time

Only one (observation 6) started on time. The lecturer was in the class before scheduled time waiting for students. However, nine of the classes started late. The lateness ranged from 10 min (observations 2 and 7) to 44 min (observation 3). In one case (observation 5), the lecturer was on time but students were not available because they were moving immediately from another lecture. In other words, the ending time from the other lecture overlapped with the starting time of the new lecture. In another case (observation 8), the lecturer was engaged in an analogous official duty and therefore reported late.

#### 6.1.2 Subtheme B: Creating a conducive and participatory environment

Some attitudes demonstrated by the educators appeared to have encouraged collegiality. For example, one lecturer's statement, "no answer is wrong, it could only be a right answer to a different question" (observation 2) caused students to participate in the teaching/learning process, which is consistent with CT teaching strategies. Also, some lecturers demonstrated a good sense of humour that was appreciated by students. For example, in observation 3, the lecturer asked a question and after the question, jokingly said, "my question to those in spectacles", which generated laughter from the students. The same lecturer appeared receptive to students’ views—allowed students to disagree with his views and even thanked students for asking questions. These strategies also demonstrated modelling of open‐mindedness (an attribute of CT) on part of the educators.

Active participation in the teaching and learning process is required to facilitate CT skills of students. However, some actions taken by some lecturers appeared to have resulted in students not fully participating in the learning process. For example, students appeared tense or nervous after the lecturer made the statements that "they [students] must respect and not make offensive statements; some of you are still adolescents. You must respect, I have always told you" (observation 8). This statement was in reaction to a comment from a student that the lecturer found to be offensive.

#### 6.1.3 Subtheme C: Teaching methods and styles

The most frequent teaching method used was student presentation. In one case (observation 6), students were given case studies from which they were requested to draw a plan of care. However, students themselves used lectures whilst presenting. General discussions followed students' presentations led by the lecturer. The presentation encouraged students to share their views freely. However, during student presentations, several students appeared disinterested and were passive in the process. Some presenters just read from the power point slides verbatim. In cases where lecturers taught, they often used the lecture method interspersed with periods of questions and answers (observations 2, 3, 4 and 8).

In one lecture (observation 3), the lecturer related lessons to real life situations (stories from the clinical settings) that appeared to have sustained the interest of the students. The lecturer also frequently moved up and down the aisles during the class session. These actions appeared to have caused students to be more attentive (which enhances memory) throughout the session.

#### 6.1.4 Subtheme D: Managing the class

Management of the class appeared to be challenging to some lecturers. For example, in observation 10, the lecturer did not act even when students were engaged in distractive behaviours. Most students generally appeared interested in the lesson. However, several students appeared indifferent with what was happening, and some conversed throughout the session (observation 10).

### 6.2 Theme II Students' characteristics

Students’ characteristics refer to actions and inactions of the students during observations that might either enhance or inhibit students’ positive learning experiences towards the acquisition of CT skills. Two sub‐themes under this theme were identified, namely distractive student behaviour and punctuality.

#### 6.2.1 Subtheme A: Distractive student behaviour

Attention/perception processing is needed to enhance short‐term memory, which leads to long‐term memory (Dwyer et al., 2014). In all classes observed, several students were engaged in distractive behaviours that might hinder memory, namely fidgeting with phones, beeping/ringing phones, petty chatting and whispering—especially those sitting at the back roll of the class. However, what appeared to be the source of most distractive behaviour—the mobile phone—was useful in helping students in some of the presentations. Students sitting in front appeared more attentive. Movement of lecturers up and down the aisles appeared to limit distractive behaviours.

#### 6.2.2 Subtheme B: Punctuality

Students arrived to lectures late. For example, during observation 2, approximately 70 students were late, with some more than 1 hr late. Also, another class session began with 62 students and ended with 117 (about 55 students late). In another instance, at a pre‐scheduled time, only 29 students were present. One student came after about 1 hr 21 min (observation 5), whilst some students left before the classes concluded.

### 6.3 Theme III: University‐wide factors/administrative support

University‐wide factors/administrative support relate to administrative factors in the university or school that might either enhance or inhibit students’ positive learning experiences towards the acquisition of CT skills. Three sub‐themes under this theme were identified: class size; scheduling of classes; and classroom layout and equipment.

#### 6.3.1 Subtheme A: Class size

Class sizes observed for the 10 sessions ranged from 34–162 with an average of 95 students. Most classes (7) were above 90 students.

#### 6.3.2 Subtheme B: Scheduling of classes

Some students who were to have a lecture immediately after the session were packed at the entrance to the lecture hall whilst engaging in conversation apparently causing distraction (observation 4). Also, some lectures started immediately after a lecture had ended with no time to move from one lecture hall to another.

#### 6.3.3 Subtheme C: Classroom layout and equipment

Classrooms’ arrangements/layouts are rectangular with desks and chair bolted down. Most ceiling mounted projectors in classrooms were dysfunctional forcing lecturers to use movable projectors which were placed too close to screens. This made power point font sizes small. Some screens were torn and dirty making projected content unclear (observation 3). Also, some public address systems were dysfunctional, and therefore, some students could not hear the lecturers. For example, during a lecture (observation 4) on three different occasions, students drew the attention of the lecturer to the fact that they could not hear him. At a point, rain stopped the lecture because students could not hear the lecturer.

## 7 DISCUSSION

Based on the observation of classroom environment in relation to instructional activities, several factors need to be considered to provide students with the desired learning experiences to the development of their CT skills. Educators’ positive behaviour which served as factors towards the enhancement of CT skills of students identified in this study is worth noting. These factors including educators’ good sense of humour and open‐mindedness appeared to inspire students to engage in the teaching–learning process were encouraging. The learning and learning process were also made entertaining. This finding is consistent with Ulloth's (2002) study which found humour to be useful in holding students’ attention, relieving anxiety, establishing rapport and making learning fun. Froneman, Du Plessis, and Koen's (2016) study on student–educator relationship identified similar characteristics needed for meaningful learning experiences of students. Similarly, other studies (Mangena & Chabeli, 2005; Raymond & Profetto‐McGrath, 2005; Raymond et al., 2018) buttress the need for nurse educators to create a conducive environment for students to develop CT skills.

Another finding worth highlighting in this study was negative educators’ factors such as being unfriendly in correcting students, using inappropriate teaching methods and poor class management skills. Similar factors were identified among educators in South Africa (Mangena & Chabeli, 2005) and Canada (Raymond & Profetto‐McGrath, 2005; Raymond et al., 2018). Mangena and Chabeli (2005) found that educators’ lack of knowledge, inappropriate teaching and assessment methods and educators’ negative attitude as barriers to the facilitation of CT skills of students.

A further noteworthy finding is the inappropriate implementation of CT teaching methods by educators. Notably, the incongruous implementation of students’ presentation and discussion methods need to be highlighted. This finding is similar to Boso and Gross' (2015) study among nurse educators in Ghana and inappropriate teaching and assessment methods identified in Mangena and Chabeli’s (2005) study.

Students’ lateness to lectures (lack of punctuality) identified in this study is worth highlighting. This finding indicates loss of valuable time which may be needed to engage with the content which may hinder the development of students’ CT skills. Also, students’ lateness to lectures appears to correspond with educators’ own late start to lectures. This appears to agree with Jack, Hamshire and Chambers' (2017) findings which highlight the influence of educator's behaviour on students. This is similar to Cruess, Cruess, and Steinert (2008) and Billings and Halstead's (2005) assertions about role modelling.

Another important finding of this study was students’ distractive behaviour. Some students engaging in distractive behaviours are not unexpected, but the degree and extent of these distractive behaviours were unanticipated. Shell (2001) identified students’ behaviour as the highest barrier to the development of CT skills of students. Also, this finding may be indicative of nurse educators’ apparent lack of appropriate classroom management skills required for meaningful learning experience of students. For example, as seen in this study, educator's movements up and down the aisles aided in the minimization of distractive behaviours of students.

The use of mobile phone during class time as an example of distractive behaviours is worth highlighting. These students may have been engaged on social media platforms such as WhatsApp, Facebook and Twitter during class sessions underscoring the penetration of social media into every facet of the students’ lives. The risk of the use of technology or social media has been noted (Ferguson, 2013). Inappropriate use of social media by students found in this study may highlight the absence of social media guidelines for students and educators. Peck (2014) suggests a purposeful use of social media to improve learning. Schools of higher learning have used social media to improve connectedness, increase access to academic libraries, create virtual classrooms and create student learning experiences to achieve desired academic outcomes (Peck, 2014). Evidence supports increased knowledge and flexibility of learning when technology is introduced into the classroom such as blended‐learning (Strickland, Gray, & Hill, 2012) and flipped‐classroom (Missildine, Fountain, Summers, & Gosselin, 2013) approaches. A purposeful use of social media should reflect the availability of social media guidelines/policy, which will likely minimize the risk or abuse of social media use.

Large class sizes were observed in this study. Pressure to increase student intake appears to overwhelm the school's capacity in terms of space and the number of qualified nurse educators at post (Bell et al., 2013; Hornsby, Osman, & Matos‐Ala, 2013). This is similar to Raymond and Profetto‐McGrath, (2005) and Raymond et al.’s (2018) studies that highlight environmental factors that influence facilitation of CT in a school. The large class sizes appear to influence teaching methods/strategies (Hornsby et al., 2013) that could be adopted by educators as identified in Gibbs, Lucas, and Spouse's (1997) study.

Another finding of this study was scheduling of classes. Classes were sometimes beginning immediately after another for the same students. This was partly accounting for late arrival of students to the next class. The late arrival of students to class may reduce their contact hours and may influence the introduction of the appropriate learning methods/strategies. Given that found time as a factor in CT development of students, reduced contact hours could inhibit the facilitation of CT of the students.

Issues relating to classroom features were observed in this study. These findings primarily relate to logistical and design issues. Logistical issues included dysfunctional ceiling mounted projectors, torn/dirty screens and inadequate public address systems. These logistical constraints may impede meaningful learning experience and consequently hinder the development of CT skills acquisition of students. This finding is consistent with other reports on challenges in the Ghanaian nursing educational system (Bell et al., 2013; Talley, 2006; Wilmot et al., 2013). The traditional rectangular classroom physical layouts with desks and chairs bolted down is inconsistent with CT skills tenets which require that physical features of classrooms involve small or large circle arrangements to allow for students to make eye contact with each other and the educator to facilitate open dialogue (Billings & Halstead, 2005).

## 8 LIMITATIONS

Given that this was a direct non‐participatory observational study, some observations might have been missed by the researchers (Creswell & Poth, 2018; Patton, 2015), especially when an attempt to minimize interruption of the teaching process, the observers of this observational study positioned themselves at the back of the classroom throughout each session. Also, there could have been observer bias. As noted by Creswell and Poth, there could have been impression management and potential deception on the part of the participants, especially the educators which might have influenced the data obtained. Several observations (10) were purposefully conducted to minimize this potential Hawthorne effect. In addition, some accounts might have been misinterpreted. However, this was minimized to some extent by reviewing the accounts with participants involved in the study.

## 9 RECOMMENDATION

Based on this study, further studies are recommended. The exploration of the experiences of nursing students and educators of instructional practices towards the development of CT skills in Ghana is highly recommended. Both quantitative and qualitative studies on how social media or technology in general could be used to facilitate meaningful learning are recommended.

This study also has implications for nurse educators and nursing school authorities who need to create a conducive environment for students for CT skills of students. Nurse educators should examine their own instructional methods/strategies with the view to adopting appropriate CT methods. In this regard, educators should aim at making learning fun and enjoyable. Educators should see themselves as role models to students regarding the demonstration of CT skills. School authorities should institute continuous faculty development programmes to help educators update their teaching skills regarding CT skills of students. School managers should provide the needed logistics needed for meaningful learning and commensurate to learning space, available faculty and other resources.

## 10 CONCLUSIONS

This study sought to observe instructional practices that influence the acquisition of CT skills of students in a classroom environment. The findings suggest that the educators’ teaching strategies have influence on learning atmosphere for CT skills facilitation of students. Also, several inhibiting and enhancing factors relating to students, university‐wide/administrative support were identified. It is therefore important that inhibiting factors are minimized or removed and enhancing factors are maintained or accentuated to help students engage in meaningful and purposeful learning experience with the view of developing their CT skills. Particularly, the role of the educators must be stressed to ensure that a conducive and participatory environment is created for student learning.

## CONFLICT OF INTEREST

We do not have any conflict of interest to report.

## AUTHOR CONTRIBUTIONS

CMB, ASVDM and JG: Conceptualization and designing of the study. CMB: Data collection, analysing and drafting of the manuscript. ASVDM and JG: Study supervision and made critical revisions on the paper. All the authors made substantial contributions to the manuscript.
